# Advanced mechanical properties of amphiphilic polymer conetworks through hierarchical reinforcement with peptides and cellulose nanocrystals†

**DOI:** 10.1039/d4py01283f

**Published:** 2025-04-25

**Authors:** Sara T. R. Velasquez, Daseul Jang, Jessica Thomas, Patrick Grysan, LaShanda T. J. Korley, Nico Bruns

**Affiliations:** a Department of Pure and Applied Chemistry, University of Strathclyde Thomas Graham Building 295 Cathedral Street Glasgow G1 1XL UK; b Department of Chemistry and Centre for Synthetic Biology, Technical University of Darmstadt Peter-Grünberg-Straße 4 64287 Darmstadt Germany nico.bruns@tu-darmstadt.de; c Department of Materials Science and Engineering, University of Delaware 127 The Green 209 DuPont Hall Newark DE 19716 USA; d Materials Research and Technology, Luxembourg Institute of Science and Technology 5 Avenue des Hauts-Fourneaux Esch-sur-Alzette L-4362 Luxembourg; e Department of Chemical and Biomolecular Engineering, University of Delaware 150 Academy Street Newark DE 19716 USA

## Abstract

Amphiphilic polymer conetworks (APCNs) have been explored for various applications, including soft contact lenses, biomaterials, and membranes. They combine important properties of hydrogels and elastomers, including elasticity, transparency, and the capability to swell in water. Moreover, they also swell in organic solvents. However, their mechanical properties could be improved. We developed a two-level, bio-inspired, hierarchical reinforcement of APCNs using cellulose nanocrystals (CNCs) to reinforce peptide-reinforced APCNs formed from hydrophobic poly-β-benzyl-l-aspartate-*block*-polydimethylsiloxane-*block*-poly-β-benzyl-l-aspartate (PBLA-*b*-PDMS-*b*-PBLA) triblock copolymer crosslinkers and hydrophilic poly(2-hydroxyethyl acrylate) (PHEA) chain segments. Bio-inspired peptide–polymer hybrids combine the structural hierarchy often found in natural materials with synthetic macromolecules, such as block copolymers with soft and hard segments, to enhance their mechanical properties. On the other hand, CNCs provide an additional means to dissipate mechanical energy in polymeric materials, thereby enhancing reinforcement. The key to homogeneously incorporating CNCs into the APCNs is the combination of hydrophobic CNCs (HCNCs) with peptide-blocks in the APCNs, exploiting the hydrogen bonding capability of the peptides to disperse the HCNCs. The effect of HCNCs on the ability of APCNs to swell in water and organic solvents, as well as on their thermal and mechanical properties, was characterized. Additionally, the nanostructure of the materials was analyzed *via* small-angle X-ray scattering (SAXS) and wide-angle X-ray scattering (WAXS). The swellability of the HCNC-containing APCNs was independent of the HCNC concentration, and all samples were highly transparent. The ideal HCNC concentration, in terms of maximal stress, strain, toughness, and reinforcement, was found to be between 6 and 15 wt%. An increase in Young's modulus of up to 500% and toughness of up to 200% was achieved. The hierarchical reinforcement also greatly strengthened the APCNs when swollen in water or *n*-hexane. Thus, HCNCs and peptide segments can be used to reinforce APCNs and to tailor their properties.

## Abstract 2

Amphiphilic polymer conetworks were mechanically reinforced with a combination of peptide blocks and cellulose nanocrystals, resulting in transparent, water-swellable and hexane-swellable gels with excellent mechanical properties.

## Graphical abstract

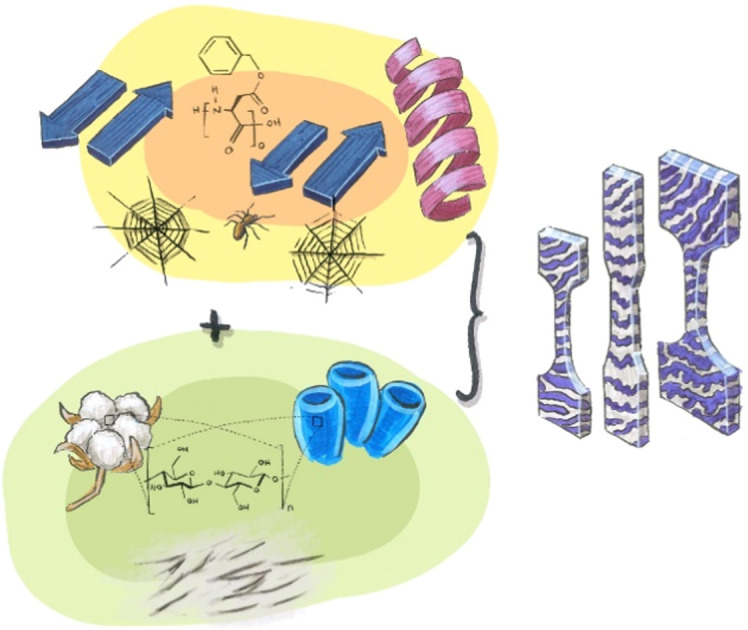

Nature is well known for creating strong and tough materials, which are typically two mutually exclusive mechanical properties in synthetic materials.^1^ This is achieved through hierarchical physical or chemical reinforcement over several length scales.^2^ The reinforcement concepts found in nature can be abstracted and applied in a bio-inspired manner to enhance man-made materials, making them better, stronger, and more sustainable. For example, strong and tough hydrogels are needed to withstand the load requirements of different applications such as contact lenses,^3–5^ biomaterials,^6–9^ and drug delivery materials.^10,11^ The mechanical properties of hydrogels can be enhanced, *e.g.*, by increasing the cross-linking densities through chain entanglements,^12^ by applying various crosslinking chemistries,^13–15^ by tailoring the network architecture,^16^ or by combining ionic physical crosslinks with covalent crosslinks in a polymer network.^17^ A very versatile method to enhance the mechanical properties of polymers is to incorporate nanoscale fillers of high mechanical strength and high aspect ratio into the material.^18,19^ Haraguchi and Takehisa pioneered nanocomposite hydrogels, in which exfoliated clay platelets were interconnected with polymer chains, resulting in highly stretchable, optical transparent hydrogels.^20^ Beyond clay, other suitable reinforcement fillers for hydrogels include metal nanoparticles,^21,22^ glass fibers,^23^ carbon nanotubes,^24^ cellulose whiskers,^25^ cellulose nanofibers (CNF),^26^ and cellulose nanocrystals (CNCs).^18,19,27–32^ CNCs, in particular, are good reinforcements for polymeric materials due to their remarkable properties, such as high mechanical strength, high aspect ratio, controllable surface chemistry, renewability and biodegradability.^18,33^ CNCs are obtained from renewable resources, including cotton, wood, or other cellulosic materials, and typically have a very high tensile strength, around 2 to 6 GPa, and a Young's modulus of 20 to 150 GPa.^26,27,34^ Depending on their source and preparation method, CNCs have a diameter that ranges between 5–70 nm and a length of 100–250 nm, *i.e.*, their aspect ratio is high.

Amphiphilic polymer conetworks (APCNs)^35–39^ represent a type of hydrogel that exhibits superior mechanical properties compared to conventional gels, as their hydrophobic component strengthens the material when swollen in water. Their most prominent market application is in silicone hydrogel soft contact lenses.^40^ Moreover, they have been explored, *e.g.*, as implant materials, biocatalyst supports, separation membranes, self-sealing breathable membranes, and microcapsules.^41–45^ Even though APCNs are stronger and tougher than most other hydrogels, their applications would benefit from improved stiffness and toughness, especially when swollen. Although studies aimed at enhancing the mechanical properties of APCNs are limited, research in this area has increased in recent years. Dynamic covalent bonds and triblock copolymer micelles have allowed for high stretchability.^46^ Another example is the use of a mechanical “fuse link” consisting of aggregated hydrophobic chain segments in a hydrophilic polymer network matrix.^47^ APCNs with dynamic covalent bonds, such as on poly(ethylene glycol)-*block*-poly(propylene glycol)-*block*-poly(ethylene glycol) (PEG-*b*-PPG-*b*-PEG) crosslinked with a triacylhydrazide oligo(ethylene glycol) triarm star cross-linker,^48^ or four-armed star block copolymers comprising PEG peripheral blocks and PPG internal blocks linked with dynamic covalent acylhydrazone bonds,^49^ have remarkable mechanical properties (such as stretchability up to 2400%^48^), as well as self-healing properties. Moreover, Tsalikis *et al.* have conducted studies using dissipative particle dynamics simulations to investigate the self-assembled bulk morphologies and mechanical properties of model APCNs, providing valuable insights, which will be very useful for the development of next-generation APCN materials.^50^

We have previously demonstrated that poly-β-benzyl-l-aspartate (PBLA) peptide segments adjacent to the crosslinking points in APCNs improve the mechanical properties of APCNs.^35^ Depending on the peptide length, they either form α-helices or β-sheets as secondary structure and efficiently reinforce polymeric materials.^51–58^ The peptides enhanced the mechanical properties of poly(2-hydroxyethyl acrylate)-*linked by*-poly(dimethylsiloxane) (PHEA-*l*-PDMS) APCNs, inducing up to a 40-fold increase in maximal stress and a 340% increase in strain when dry, relative to the base APCNs.

Here, we extend these results by introducing a second level of reinforcement into peptide-reinforced APCNs by incorporating CNCs. However, the hydrophilic nature of CNCs is a major drawback in the synthesis of CNC-reinforced APCNs, as the nanocrystals are difficult to disperse homogeneously in the hydrophobic monomer mixture used in the synthesis of PHEA-*l*-PDMS APCNs. The CNCs tend to form aggregates that are inefficient in reinforcing the material. Certain modification techniques, such as carbamation, esterification, and silylation, have been used to increase the hydrophobicity of CNCs, thereby rendering them miscible with hydrophobic monomers, solvents, or polymers.^19,59^ For example, 2-ureido-4[1*H*]pyrimidinone (UPy)-modified CNCs allowed the production of nanocomposites based on poly(ethylene), polystyrene-*b*-polybutadiene-*b*-polystyrene elastomers and poly(ethylene oxide-*co*-epichlorohydrin), with increased stiffness and strength, while still maintaining a high strain-at-break.^18^

Here, we use hydrophobically modified cellulose nanocrystals (HCNCs) to prepare HCNC-reinforced APCNs based on the hydrophobic crosslinker α,ω-methacrylate functionalized PBLA_5_-*b*-PDMS-*b*-PBLA_5_ that contains β-sheet-forming PBLA peptide blocks, which provide additional physical crosslinks through hydrogen bonding and enhance the mechanical properties of the material.^35^ This crosslinker was used to crosslink hydrophobically masked trimethylsilyl 2-hydroxyethyl acrylate (TMS-HEA), yielding hydrophobic precursor networks that were then converted into PHEA-*l*-(PBLA_5_-*b*-PDMS-*b*-PBLA_5_) APCNs by cleaving off the TMS groups. The bio-inspired, two-level, hierarchical reinforcement of APCNs resulted in APCNs that were reinforced in both the dry and water-swollen states.

## Results and discussion

### Synthesis of peptide-reinforced amphiphilic polymer conetworks

The APCNs were synthesized by UV-induced radical polymerization, as described in the Experimental section. They were prepared using dimethylacetamide (DMAc) as a solvent for a homogeneous dispersion of the HCNCs, whereby the minimum amount of solvent required to disperse the HCNCs was determined experimentally. The HCNC- and peptide-reinforced samples were compared to HCNC-free peptide-containing APCNs that were prepared with the same amount of DMAc in the monomer mixture.

The concentration of the HCNCs was varied between 0 and 22 wt% in the final networks, while the ratio of TMS-HEA to crosslinker was kept constant to obtain PHEA-*l*-(PBLA_5_-*b*-PDMS-*b*-PBLA_5_) APCNs with a 1 : 1 wt% ratio of hydrophobic to hydrophilic phase (Table 1). For brevity, HCNC-peptidic APCNs were designated as HCNC_XX, where XX represents the weight fraction of HCNC in weight percent (%).

**Table 1:** Composition of the reaction mixtures used to synthesize PHEA-*l*-(PBLA_5_-*b*-PDMS-*b*-PBLA_5_) APCNs with 0 wt% to 22 wt% of HCNCs

	Composition of monomer mixture (mg)		HCNC (mg)	Solvent DMAc (μl)
	MA-PBLA_5_-*b*-PDMS-*b*-PBLA_5_-MA	TMS-HEA		
HCNC_00	250	405	0	300
HCNC_01	250	405	6.6	300
HCNC_03	250	405	20.3	300
HCNC_06	250	405	41.9	300
HCNC_09	250	405	64.8	300
HCNC_12	250	405	89.4	300
HCNC_15	250	405	115.7	300
HCNC_18	250	405	143.9	300
HCNC_22	250	405	184.9	300

### Analysis of the optical transparency

**Fig. 1:**
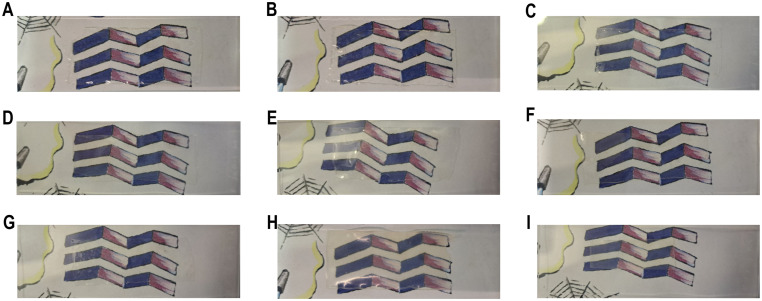
Characterization of the transparency of dry HCNC-reinforced PHEA-*l*-(PBLA_5_-*b*-PDMS-*b*-PBLA_5_) APCNs by photographs of the samples positioned on a microscope slide over a printed background, which shows a drawing of a purple beta sheet structure and some other graphical elements. The samples were placed over the purple beta sheet structure. (A) HCNC_00, (B) HCNC_01, (C) HCNC_03, (D) HCNC_06, (E) HCNC_09, (F) HCNC_12, (G) HCNC_15, (H) HCNC_18, (I) HCNC_22. (See Fig. S1† for an example where the location of an APCN is highlighted.)

A key property of APCNs is their transparency.^3,60^Fig. 1 shows optical photographs of the APCNs placed over a printed image, revealing that all the HCNC-reinforced APCNs were fully transparent, with the underlying image remaining clearly visible. Transparency indicates that the HCNCs are well dispersed in the polymeric material and that the HCNCs (5–20 nm wide and 150–250 nm long) do not form aggregates that scatter light even at HCNC concentrations as high as 22 wt%. Notably, these results indicate that peptide- and HCNC-reinforced APCNs may be suitable for applications where transparency is required, such as in soft contact lens materials.

### Thermal analysis

**Fig. 2:**
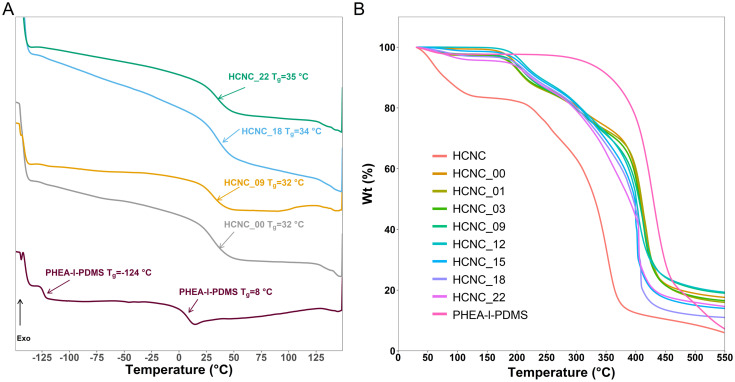
Characterization of the thermal properties of CNC-reinforced PHEA-*l*-(PBLA_5_-*b*-PDMS-*b*-PBLA_5_) APCNs, and for comparison of PHEA-*l*-PDMS. (A) DSC curves, with arrows indicating the glass transition temperature of each sample. (B) TGA thermograms.

The phase separation of APCNs can be investigated using differential scanning calorimetry (DSC) to determine if there are two glass transition temperatures (*T*_g_) corresponding to the individual polymers. While conventional PHEA-*l*-PDMS APCNs have two glass transition temperatures, at −124 °C and 8 °C corresponding to PDMS and PHEA, respectively, the peptide-reinforced APCNs had a single *T*_g_ between 32–35 °C, independent of whether HCNCs were incorporated into them or not (Fig. 2). This result is consistent with our previous report, in which dynamic mechanical analysis (DMA) data showed that peptide-reinforced APCNs only exhibit a single *T*_g_ despite their nanophase-separated morphology.^35^ Most likely, this behavior is due to miscibility between the PBLA blocks and PHEA, and the mobility of the PDMS chain segments being inhibited by the adjacent peptide blocks.^35^ The presence of HCNCs in the APCNs did not significantly influence the *T*_g_. Moreover, increasing the HCNC concentration did not alter the *T*_g_ of the APCNs, most likely because the HCNCs are not covalently bound to the polymer or strongly interact with it, therefore not affecting the mobility of the chain segments.

To establish the thermal stability of the APCNs and gain insight into their thermal degradation, thermogravimetric analysis (TGA) was carried out (Fig. 2B). For comparison, TGA was also performed on PHEA-*l*-PDMS APCNs and pure HCNCs. The peptidic APCN without incorporated HCNC degraded in two steps with onsets at 185 °C, tentatively linked to the peptide blocks, and 380 °C, corresponding to the degradation of PHEA and PDMS chain segments (*vide infra*). Small amounts of HCNCs, *i.e.*, up to 3 wt%, did not affect the thermal decomposition profile of the APCNs. With increasing HCNC content, the contribution from the CNCs became noticeable, showing slight weight loss below 110 °C, probably due to the loss of water adsorbed on the surface of the HCNCs, followed by the onset of degradation at 180 °C. As a result, the main degradation of the HCNC-reinforced peptidic APCNs with ≥9 wt% HCNCs was between 300 °C and 410 °C, most likely due to a combination of HCNC and PHEA/PDMS weight loss. By contrast, PHEA-*l*-PDMS was thermally stable until ∼350 °C, and the TGA trace shows one major degradation step, attributed to the degradation of the PHEA/PDMS conetwork.

### Morphology analysis

**Fig. 3:**
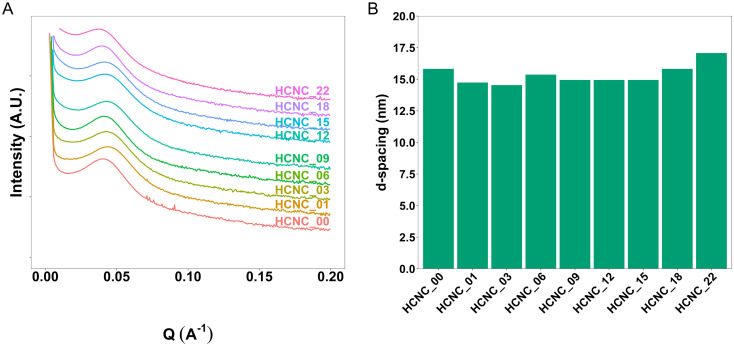
Characterization of the morphology of dry CNC-reinforced PHEA-*l*-(PBLA_5_-*b*-PDMS-*b*-PBLA_5_) APCNs. (A) SAXS profiles. (B) SAXS-derived domain-domain correlation *d*-spacing.

Small-angle X-ray scattering (SAXS) provides information about the phase-separated morphology of APCNs and was used to characterize bulk samples in the absence of solvents.^61^ The position of the main peak (q*) in the SAXS traces allows determining the *d*-spacing, *i.e.*, the average distance of the scattering domains (Fig. 3). The HCNCs in the APCN matrix had minimal influence on the domain size and phase separation. The *d*-spacing of the peptidic APCN without HCNCs was 15.8 nm. The *d*-spacing decreased to 14.7 nm with 1 wt% HCNC and remained relatively constant, with minimal variation, until an HCNC content of 18 wt% and 22 wt%, at which it increased to 15.8 nm and 17.1 nm, respectively. This change in *d*-spacing indicates that at high concentration, the HCNCs widen the domains of the APCNs.

**Fig. 4:**
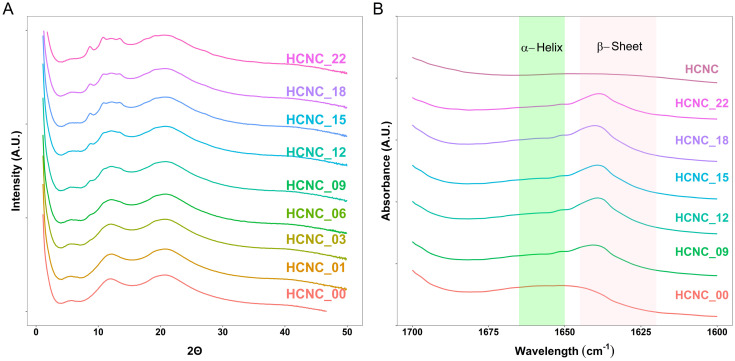
Morphological characterization of HCNC-reinforced PHEA-*l*-(PBLA_5_-*b*-PDMS-*b*-PBLA_5_) APCNs. (A) WAXS data. (B) ATR-FTIR spectra of APCNs in the amide I region.

Wide-angle X-ray scattering (WAXS) and attenuated total reflection Fourier transform infrared (ATR-FTIR) spectroscopy were used to confirm the presence of peptide β-sheets. The WAXS results (Fig. 4A) show peaks at 2*θ* ≈ 12° and 22° corresponding to the PDMS and PHEA phases, respectively. The peptides form β-sheets, resulting in a WAXS peak at 2*θ* ≈ 5° (*q* = 0.36 Å^−1^)^62–64^ observable in all the investigated peptidic APCNs. Moreover, the ATR-FTIR spectra reveal β-sheet peaks for all the samples (Fig. 4B).^35^ Thus, the presence of HCNCs did not impact the formation of peptide β-sheets. With increasing HCNC content, starting from 3 wt%, a WAXS peak at 2*θ* ≈ 8° emerged and became stronger through to 22 wt%. The peak from the PDMS at 2*θ* ≈ 12° also changes, becoming broader with two smaller peaks appearing at higher HCNC concentrations. This observation confirms the presence of the HCNCs in the APCNs, as the crystalline microstructure of the HCNCs results in two peaks at 2*θ* ≈ 4.5° and 16.5°.^65^ However, due to the PDMS peak, peak shifting may have occurred.

**Fig. 5:**
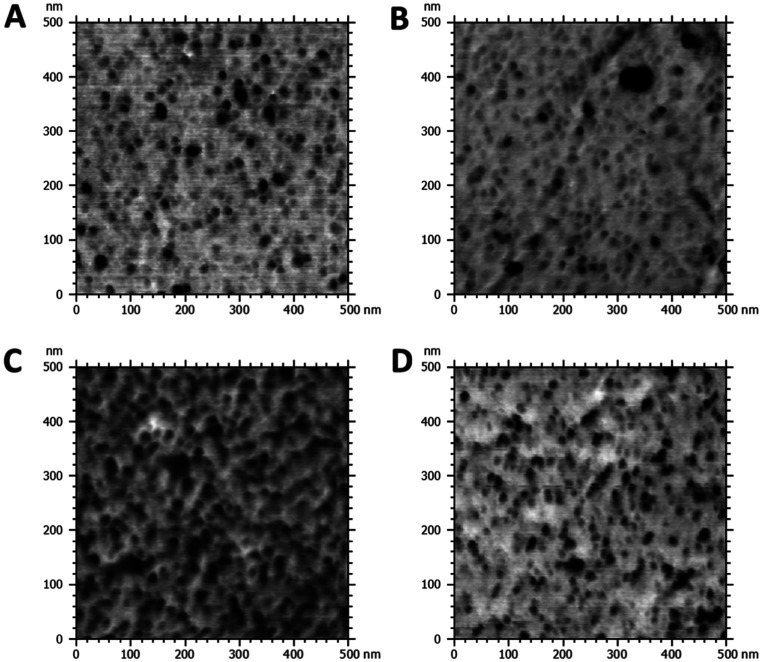
AFM phase mode images of cross-sections of HCNC-reinforced PHEA-*l*-(PBLA_5_-*b*-PDMS-*b*-PBLA_5_) APCNs. (A) HCNC_00, HCNC_09, HCNC_18, HCNC_22.

APCNs typically exhibit a phase-separated morphology at the nanoscale, characterized by dark soft (hydrophobic) and bright hard (hydrophilic) domains in phase-mode atomic force microscopy (AFM) images.^35^ The morphology of APCNs depends on their composition, the length of the macromonomer and ranges from spherical to bicontinuous phase morphologies.^50,66^ It should be noted that the bulk morphology of APCNs usually differs from the morphology observed on their surface because components of the monomer mixture tend to accumulate on the surface of APCNs during polymerization.^66^ AFM images in phase mode of microtomed cross-sections of the APCNs thus reveal their bulk morphology (Fig. 5). The APCNs investigated herein have a balanced 50–50 wt% composition of hydrophilic PHEA segments and hydrophobic PBLA_5_-*b*-PDMS-*b*-PBLA_5_ macrocrosslinker. However, AFM images of the HCNC_00 show round hydrophobic domains in a sponge-like hydrophilic phase, which is usually observed for APCNs where the hydrophobic polymer chain segments are the minority component.^35,67,68^ A possible explanation is that the peptide segments are less hydrophobic and harder than the PDMS blocks, and according to the DSC results discussed above, might mix with the PHEA. Thus, the dark domains in the AFM images are most likely only PDMS. The samples containing HCNCs show a higher content of the hard phase, which is likely composed of the HCNCs, the peptide segments, and PHEA. It is challenging to quantitatively assess the volume ratio between the APCN's hard and soft domains, as the APCNs would have to be cut with the same level of roughness to achieve comparable contrast in the cross sections.

### Swelling behavior

APCNs swell in water and nonpolar organic solvents due to their amphiphilic nature and phase separation into hydrophobic and hydrophilic domains at the nanoscale.^60^ To analyze the effect of the HCNCs on the swelling, the swelling ratio (*S*_vol_) was measured by incubating the samples at room temperature in water and *n*-hexane. PHEA-*l*-(PBLA_5_-*b*-PDMS-*b*-PBLA_5_) APCNs without HCNCs swelled to *S*_vol_ = 3.13 ± 0.8 and 1.23 ± 0.2 in water and the organic solvent, respectively. The swelling ratio of these samples differs between the two solvents, despite the 50–50 ratio of hydrophobic to hydrophilic components, possibly because the hydrogen bonds of the peptides are disrupted when the samples are swollen in water but not in *n*-hexane. This swelling is significantly higher than that of similar APCNs investigated in our previous study,^35^ most likely because the peptide-reinforced APCNs investigated herein were synthesized in a different solvent and at a lower monomer concentration, resulting in less entangled polymer chains and, consequently, fewer additional physical crosslinking points.^12^

**Fig. 6:**
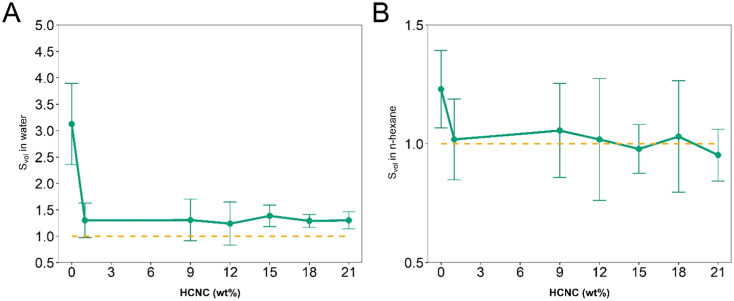
Swelling of PHEA-*l*-(PBLA_5_-*b*-PDMS-*b*-PBLA_5_) APCNs as a function of the HCNC concentration. (A) Swelling ratio *S*_vol_ in deionized water. (B) Swelling ratio *S*_vol_ in *n*-hexane (mean of *n* = 5 samples ± SD). The error bars in B appear large because the results fall within the error range of the measurement.

HCNCs significantly decreased the swellability of the APCNs at all HCNC concentrations (Fig. 6). Water swelled the HCNC-containing APCNs to *S*_vol_ = 1.3, independent of the HCNC concentration. In *n*-hexane, the APCNs exhibited minimal swelling, possibly because the HCNCs form hydrogen bonds between them, to the hydrophilic PHEA chain segments and to the peptide blocks, thus introducing additional physical crosslinking points that are not disrupted by the organic solvent.

### Mechanical properties

**Fig. 7:**
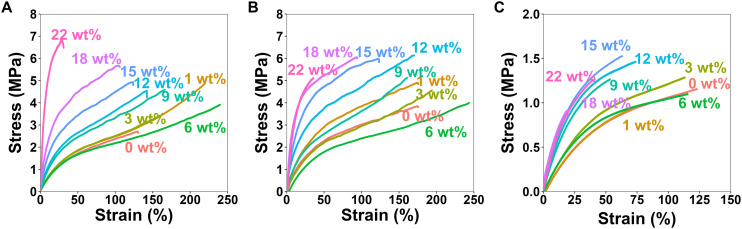
Representative stress–strain curves for the uniaxial stress–strain mechanical tests of the HCNC-reinforced PHEA-*l*-(PBLA_5_-*b*-PDMS-*b*-PBLA_5_) APCNs measured at a strain rate of 10 mm min^−1^. (A) Stress–strain curves of the dry samples with different HCNC content. (B) Stress–strain curves of samples swollen in *n*-hexane with different HCNC content. (C) Stress–strain curves of samples swollen in water with different HCNC contents.

**Fig. 8:**
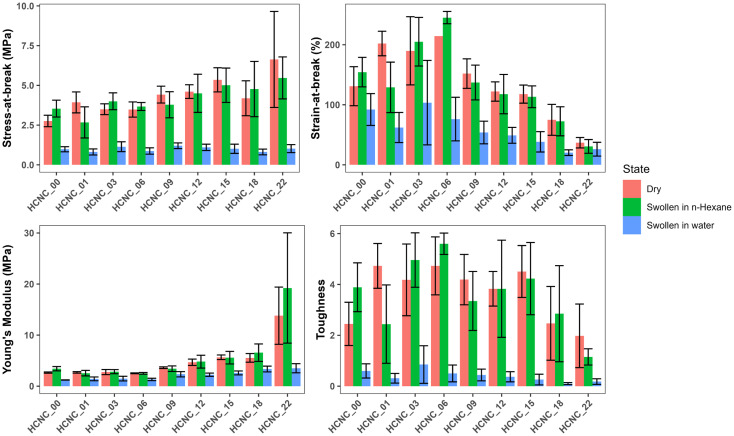
Bar graph summarizing the mechanical properties of HCNC-reinforced PHEA-*l*-(PBLA_5_-*b*-PDMS-*b*-PBLA_5_) APCNs. (A) Stress at break, (B) strain at break, (C) Young's modulus, and (D) toughness (mean of *n* = 6 samples ± SD).

To investigate how the HCNCs influence the mechanical properties of the APCNs, uniaxial tensile tests were carried out in both dry and water-swollen conditions. Representative stress–strain curves are reported in Fig. 7, and the results of the mechanical tests are summarized in Fig. 8, which includes stress-at-break, strain-at-break, Young's modulus, and toughness. These results are discussed below (see also the ESI†).

For the dry samples, there was a 2.4-fold increase in maximum stress from 2.8 ± 0.4 MPa at 0 wt% HCNCs to 6.6 ± 3.0 MPa at 22 wt% HCNCs. The strain-at-break peaked at 214 ± 33% with 6 wt% HCNCs and decreased to 37 ± 9% at 22 wt% HCNCs, which is lower than the strain-at-break of the peptide APCN without HCNCs. Young's modulus increased continuously from 2.7 ± 0.1 MPa at 0 wt% HCNCs to 14.8 ± 6 MPa for 22 wt% HCNC. The toughness of HCNC-reinforced APCNs with HCNC contents between 1 wt% and 16 wt% was approximately twice that of APCNs without HCNCs. At a higher HCNC content, the toughness was reduced to values similar to those of the APCNs without HCNCs. In conclusion, the HCNCs reinforced the APCNs and enhanced their mechanical properties in the dry state. However, above a certain loading, HCNCs did not provide a mechanical advantage, possibly because of the formation of agglomerates in the polymer, which is also observed in the reduced reproducibility of the results at high HCNC concentrations as shown in Fig. S2–S4,† where the error range increases with the increase of the HCNC concentration.

To test the mechanical properties of APCNs swollen in an organic solvent, the samples were immersed in *n*-hexane overnight and then subjected to stress–strain experiments. With increasing HCNC content, the stress-at-break increased from 3.5 ± 0.5 MPa for samples without HCNCs to 5.5 ± 1.3 MPa for samples with 22 wt% HCNCs. APCNs with 6 wt% HCNCs exhibited the highest strain-at-break (245 ± 10%), which decreased to 31 ± 11% for the highest HCNC concentration tested, and therefore fell below the strain-at-break of the APCN without HCNCs. Young's modulus increased continuously from 3.4 ± 0.4 at 0 wt% HCNCs to 19 ± 11 MPa for 22 wt% HCNCs. The highest toughness of 5.6 ± 0.4 MJ m^−3^ was measured for samples with 6 wt% HCNCs, and the lowest toughness was recorded for APCNs with 22 wt% HCNCs. In summary, the HCNCs also increased the mechanical properties of the APCNs swollen in *n*-hexane. The addition of HCNCs up to 6 wt% increased maximum stress, maximum strain, and toughness. However, higher HCNC concentrations caused a reduction in mechanical performance, except for the Young's modulus, which increased continuously with HCNC concentration. Thus, the APCNs became stiffer with increasing HCNC content.

The mechanical properties of water-swollen APCNs were consistently lower than those of samples swollen in *n*-hexane or dry samples. The stress-at-break was 1.2 ± 0.2 MPa for the 9 wt% HCNCs. It is similar for all samples up to 22 wt% HCNCs, with all values falling within the margin of error. The highest strain-at-break was recorded for the 3 wt% samples with 104 ± 70%. However, this value has a broader standard deviation compared to the other HCNC concentrations, possibly because the HCNCs are not homogeneously distributed within the sample and act as failure points. Therefore, further studies are needed for this composition. Young's modulus increased linearly with increasing HCNC content, from 1.2 ± 0.03 to 3.5 ± 0.9 MPa. The increase in HCNC concentration decreased the toughness of the water-swollen APCNs. The 18 and 22 wt% HCNC samples exhibited the lowest toughness when swollen in water. The HCNCs clearly affected the mechanical properties of the APCNs swollen in water, probably due to the formation of hydrogen bonds.

In general, HCNCs reinforced the APCNs under different conditions. According to these results, samples with HCNC content between 6 and 15 wt% appear to provide the ideal balance of mechanical behavior, exhibiting high stress- and strain-at-break, Young's modulus, and toughness. Increasing the HCNC content to values higher than 22 wt% was not possible, as the HCNCs did not disperse homogeneously.

## Conclusions

This work presents a hierarchical reinforcement strategy for APCNs. Hydrophobic peptide blocks provide a first level of reinforcement, while the HCNCs provide an additional reinforcement mode. The main properties of the APCNs were studied to analyze the effect of the HCNCs. All the samples were transparent, which is a remarkable result given that HCNCs could scatter light at high concentrations if they are not well dispersed in the polymer matrix. The swelling of the peptidic APCNs without HCNCs was higher than that of similar peptidic APCNs reported previously. However, as the content of HCNCs increased, swelling in both water and *n*-hexane was reduced and remained constant, independent of the HCNC content. Nevertheless, the HCNC-reinforced APCNs swell in water and *n*-hexane, and are, therefore, hydrogels and lyogels. The *T*_g_ of the HCNC-reinforced samples remained between 30 and 36 °C. The TGA analysis reveals a decrease in degradation temperature with the addition of peptides and HCNCs. The optimal reinforcement of dry samples and samples swollen in *n*-hexane was obtained at HCNC concentrations between 6 and 15 wt%. For the samples swollen in water, reinforcement was observed for all the HCNC samples. Therefore, HCNCs are a novel additive to reinforce APCNs. However, synthesis required the use of a solvent, which has a slightly negative effect on the mechanical properties compared to the previously reported peptide-reinforced APCNs that were synthesized with less solvent. This work demonstrates that HCNCs can serve as an additional means to reinforce APCNs without compromising the material's key properties. Thus, the HCNC- and peptide-reinforced APCNs could find applications ranging from soft contact lenses to biomaterials and membranes.

## Methods/experimental section

### General information

The APCNs were prepared through UV-induced free radical polymerization. This work focused on a 50 : 50 weight ratio of PHEA : PDMS as hydrophilic phase and hydrophobic macrocrosslinkers MA-PBLA_5_-*b*-PDMS-*b*-PBLA_5_-MA.

### Materials

All materials used for the APCNs preparation were used as received unless stated otherwise. Hydrophobically modified CNCs were purchased from Celluloselab, Canada. Bis(3-aminopropyl) terminated polydimethylsiloxane (PDMS, molecular weight = 2500 g mol^−1^), β-benzyl-l-aspartate (BLA), triphosgene, isocyanoethyl methacrylate, tin dibutyl dilaurate, 2-hydroxyethyl acrylate, triethylamine, chlorotrimethylsilane, photoinitiator bis(2,4,6-trimethylbenzoyl)-phenylphosphineoxide (Irgacure 819) and all analytical grade solvents were purchased from Sigma Aldrich. MA-PDMS-MA (viscosity 50–90 cSt, molecular weight = 4500–5500 g mol^−1^) was purchased from ABCR (Germany). Adhesive polypropylene tape (Tesafilm™, 50 μm thickness) was bought from Tesa, Germany. MA-PBLA_5_-*b*-PDMS-*b*-PBLA_5_-MA was synthesized as previously described.^35^

### Sample preparation

Table 1 summarizes the amount of reagents utilized. The hydrophobic component (MA-PBLA_5_-*b*-PDMS-*b*-PBLA_5_-MA; or for comparison, 250 mg MA-PDMS-MA) was mixed with dimethylacetamide (DMAc) and placed on a vortexer until a homogeneous mixture was obtained. HCNC powder was added to the triblock copolymer solution, and the mixture was ultrasonicated for 5 min (40% amplitude, and pulse of 15 s on, 10 s off). The addition of CNCs to the monomer mixture required an increase in the amount of solvent (and a switch from DMSO to DMAc) used for dispersing and processing of the monomer mixture when compared to our previous research.^35^ The samples were prepared with the minimum amount of solvent necessary for a homogeneous dispersion, which was determined experimentally for the highest HCNC concentration. The hydrophobically masked monomer TMS-HEA was added to the mixture, which was then vortexed for 180 s and ultrasonicated for 10 min. The UV initiator (3 mg) was added to the monomer mixture, which was vortexed for a further 90 s. Then, the monomer mixture was placed on glass slides covered with transparent polypropylene adhesive tape. The tape was also applied to the sides as a spacer. The samples were polymerized in a UV light system (Dymax 500) for 9 min, on each side by flipping the samples after one side was completed. Afterward, the APCNs were placed overnight in 200 mL of a 1 : 1 v/v ratio of water and isopropanol to cleave off the TMS groups. Finally, the materials were left in methanol for 5 hours and subsequently dried in air.

### Characterization methods

#### Swelling of APCNs

The swelling behavior of the APCNs was measured by immersing samples of approximately 3 mm × 6 mm in *n*-hexane and distilled water, respectively, overnight at room temperature. The edge length (*L*_*i*_) was measured for the dry and swollen samples by placing the samples on millimeter paper, capturing photos with a phone camera (Samsung A53), and analyzing them using ImageJ. The swelling ratio, *S*_vol_, was determined from the edges of the sample as:
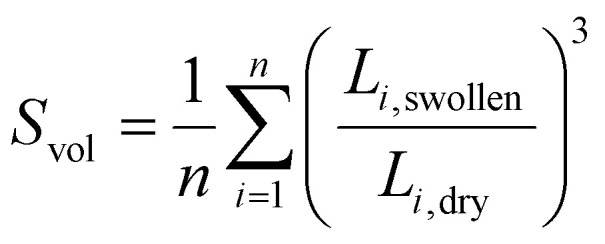
where *n* denotes the number of edges.

#### DSC

Differential scanning calorimetry (DSC) was measured using a DSC 3 Star System, Mettler Toledo. All the samples were measured for two cycles at a heating and cooling rate of 10 °C min^−1^, from −150 °C to 150 °C to −150 °C. The glass transition temperature was determined from the transition mid-point of the second heating curve.

#### TGA

Thermogravimetric Analysis (TGA) was measured using a TGA Star System, Mettler Toledo. All the samples were measured in N_2_ atmosphere (10 ml min^−1^) from 30 to 600 °C at a heating rate of 10 °C min^−1^. All the samples were normalized to weight loss %. The weight change was calculated from the step change on the slope of the curve.

#### ATR-FTIR

Attenuated total reflection – Fourier transform infrared (ATR-FTIR) spectroscopy was used to analyze the presence and content of β-sheets and α-helices and the effect of the CNCs on the secondary structures in the peptidic APCNs and peptidic triblock copolymers using a Spectrum3 spectrometer (PerkinElmer) equipped with a UATR-Diamant/ZnSe-topplate. All samples were measured in absorbance mode with 128 scans. MicroLab was used to obtain and analyze the spectra.

#### SAXS and WAXS

SAXS measurements were performed using a Xenocs Xeuss 2.0. For the measurements, X-rays were generated at 50 kV/0.6 mA at a beam wavelength of 1.542 Å (Cu Kα radiation) and sample-to-detector distances of 1200 mm and 72 mm, respectively, for SAXS and WAXS. The scattered beam was recorded on a CCD detector with a pixel resolution of 172 × 172 μm^2^. The scattering patterns of APCN films (∼1.5 × 1.5 cm^2^) were recorded over 30 min of exposure time at room temperature. 2D patterns were azimuthally integrated to obtain the scattering intensity as a function of the scattering vector, *q*, where *q* = 4π sin(*θ*)/*λ*. 2*θ* is the scattering angle. The azimuthal integration was obtained using Foxtrot 3.4.9.

#### AFM

Topography images and phase mode images were acquired in the air in tapping mode at a scan rate of 3 Hz and a resolution of 512 × 512 pixels^2^ on an MFP3D INFINITY microscope (Oxford Instrument, UK). Semi-contact silicon AC160TS AFM tips (Olympus, Japan) with a cantilever spring constant of 26 N m^−1^ were used. Surface topography was acquired by maintaining a constant first resonance amplitude in the cantilever *via* the feedback loop of the AFM acting on the piezo *Z* direction. The phase shift signal was recorded at the same time. Prior to the analysis, the APCN films were cross-sectioned and surfaced at 0 °C using a UC6 Cryo-Ultramicrotome (Leica, Germany) to obtain a representative image of the inner film composition and avoid possible skin effects. Data analysis was performed with MountainSpip software (Digital Surf, France).

#### Tensile testing

The mechanical properties were measured using a Testometric M250-2.5 CT tensile machine with a 100 N load cell. A total of six samples of each type of APCN were tested at a speed of 10 mm min^−1^. The samples were cut in dog bone shape following DIN 53504 S3 specifications with a sample thickness of ∼0.2 mm. All tests were performed for dry samples and for samples swollen in H_2_O and *n*-hexane, at temperatures between 20–23 °C and air humidity between 25–38%.

## Author contributions

S. T. R. Velasquez: conceptualization, methodology, resources, formal analysis, investigation, data curation, writing – original draft, writing – review & editing, visualization. D. Jang: investigation, writing – review & editing. J. Thomas: investigation, writing – review & editing. P. Grysan: investigation, writing – review & editing. L. T. J. Korley: conceptualization, resources, writing – review & editing, supervision, project administration, funding acquisition. N. Bruns: conceptualization, methodology, resources, writing – review & editing, supervision, project administration, funding acquisition.

## Data availability

The datasets generated and analyzed during the current study are openly available from the University of Strathclyde's Pure repository at https://doi.org/10.15129/7fc1b9a7-99d2-4666-8709-e0d319afd131.

## Conflicts of interest

The authors declare no conflict of interest.

## Supplementary Material

PY-016-D4PY01283F-s001
