# Supplementary material for: Advanced mechanical properties of amphiphilic polymer conetworks through hierarchical reinforcement with peptides and cellulose nanocrystals
Source: Polym Chem. 2025 Apr 25;16(22):2618–28. doi: 10.1039/d4py01283f (PMC12061020; doi:10.1039/d4py01283f)
Supplement: PY-016-D4PY01283F-s001 [file PY-016-D4PY01283F-s001.pdf]

Supplementary Information (SI) for Polymer Chemistry.
This journal is © The Royal Society of Chemistry 2025

Supporting Information

Advanced Mechanical Properties of Amphiphilic Polymer Conetworks through Hierarchical Reinforcement with Peptides and Cellulose Nanocrystals

*Sara T. R. Velasquez, Daseul Jang, Jessica Thomas, Patrick Grysan, LaShanda T. J. Korley, Nico Bruns**

Dr. S. T. R. Velasquez, Prof. N. Bruns

Department of Pure and Applied Chemistry, University of Strathclyde, Thomas Graham Building, 295 Cathedral Street, Glasgow G1 1XL, United Kingdom.

Department of Chemistry and Centre for Synthetic Biology, Technical University of Darmstadt, Peter-Grünberg-Straße 4, 64287 Darmstadt, Germany.

E-mail: nico.bruns@tu-darmstadt.de

Dr. D. Jang, J. Thomas

Department of Materials Science and Engineering, University of Delaware, 127 The Green, 209 DuPont Hall, Newark, DE 19716, USA.

Dr. P. Grysan

Materials Research and Technology, Luxembourg Institute of Science and Technology, 5 Avenue des Hauts-Fourneaux, Esch-sur-Alzette L-4362, Luxembourg

Prof. L. T. J. Korley

Department of Materials Science and Engineering, University of Delaware, 127 The Green, 209 DuPont Hall, Newark, DE 19716, USA.

Department of Chemical and Biomolecular Engineering, University of Delaware, 150 Academy Street, Newark, DE 19716, USA.

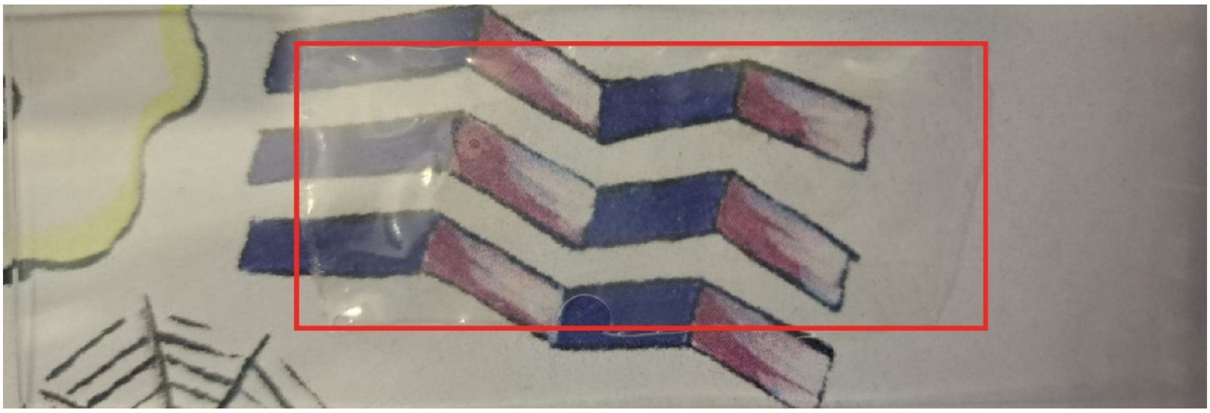

Figure S1. Photograph of PHEA-l-(PBLA$_5$-b-PDMS-b-PBLA$_5$) APCNs HCNC_09 shown in Figure 1E. The red square delimitates the area where the transparent APCNs is located.

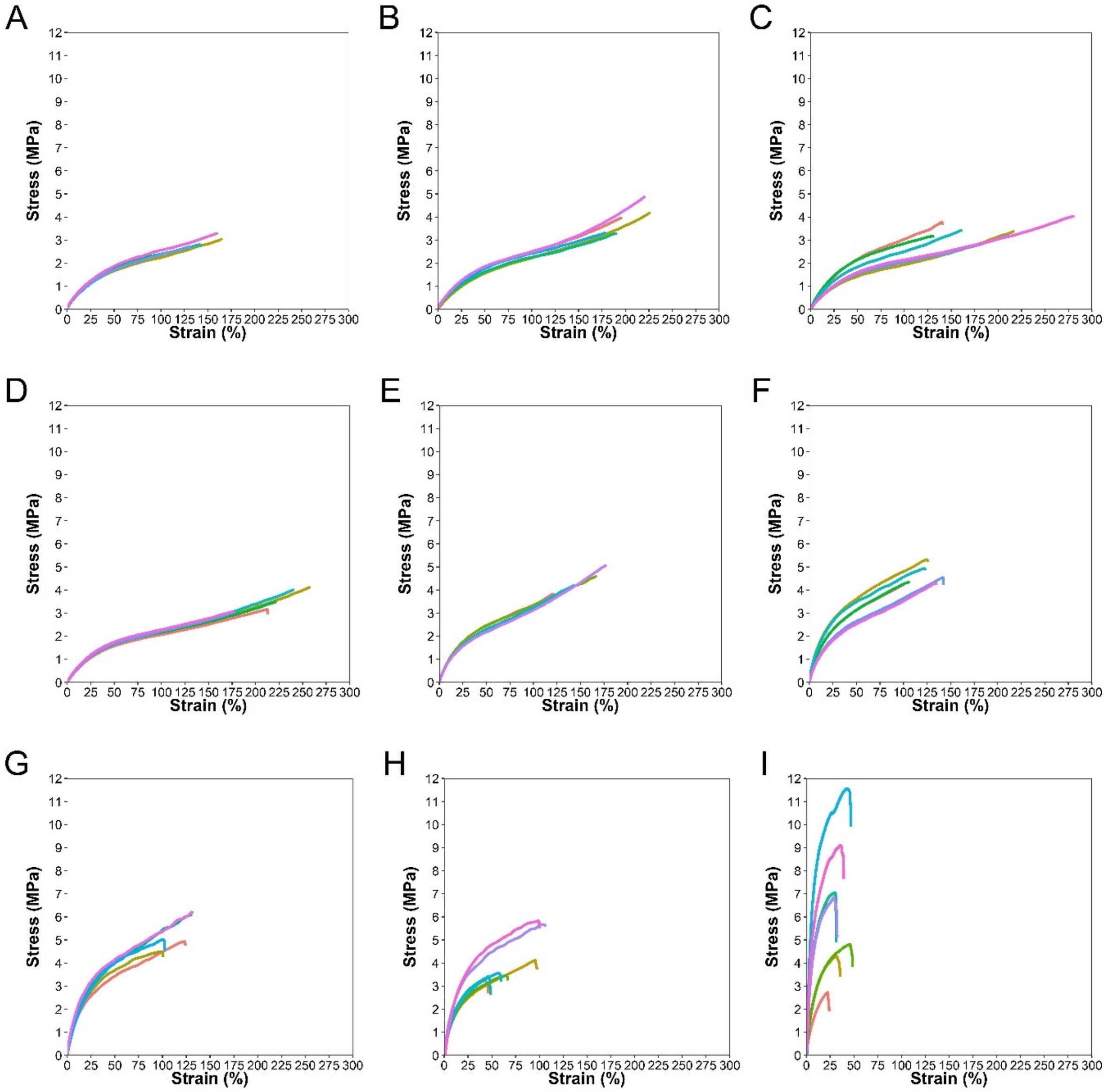

Figure S2. Stress-strain curves for the prepared samples measured in the dry state at room temperature. A) HCNC_00, B) HCNC_01, C) HCNC_03, D) HCHC_06, E) HCNC_09, F) HCNC_12, G) HCNC_15, H) HCNC_18, I) HCNC_22. All samples were measured at 10 mm min^{-1}. *(Mean of n = 6 samples ± SD).*

Table S1. Mechanical test results for the prepared samples measured in the dry state at room temperature. (Mean of n = 6 samples ± SD).

Composition	Stress-at-break (MPa)	Strain-at-break (%)	Young's Modulus (MPa)	Toughness (MJ m^{-3})
00	2.76 ± 0.36	130.92 ± 32.49	2.66 ± 0.12	2.45 ± 0.85
01	3.93 ± 0.66	202.04 ± 20.42	2.70 ± 0.15	4.73 ± 0.88
03	3.50 ± 0.34	189.90 ± 56.75	2.76 ± 0.50	4.18 ± 1.41
06	3.48 ± 0.48	214.34 ± 32.73	2.52 ± 0.08	4.73 ± 1.14
09	4.42 ± 0.53	151.93 ± 24.56	3.62 ± 0.16	4.19 ± 0.99
12	4.61 ± 0.43	122.07 ± 16.07	4.68 ± 0.62	3.83 ± 0.68
15	5.35 ± 0.76	117.78 ± 15.06	5.66 ± 0.45	4.51 ± 1.02
18	4.19 ± 1.10	74.97 ± 25.76	5.54 ± 0.85	2.47 ± 1.45
22	6.63 ± 3.02	36.85 ± 8.72	13.80 ± 5.60	1.98 ± 1.25

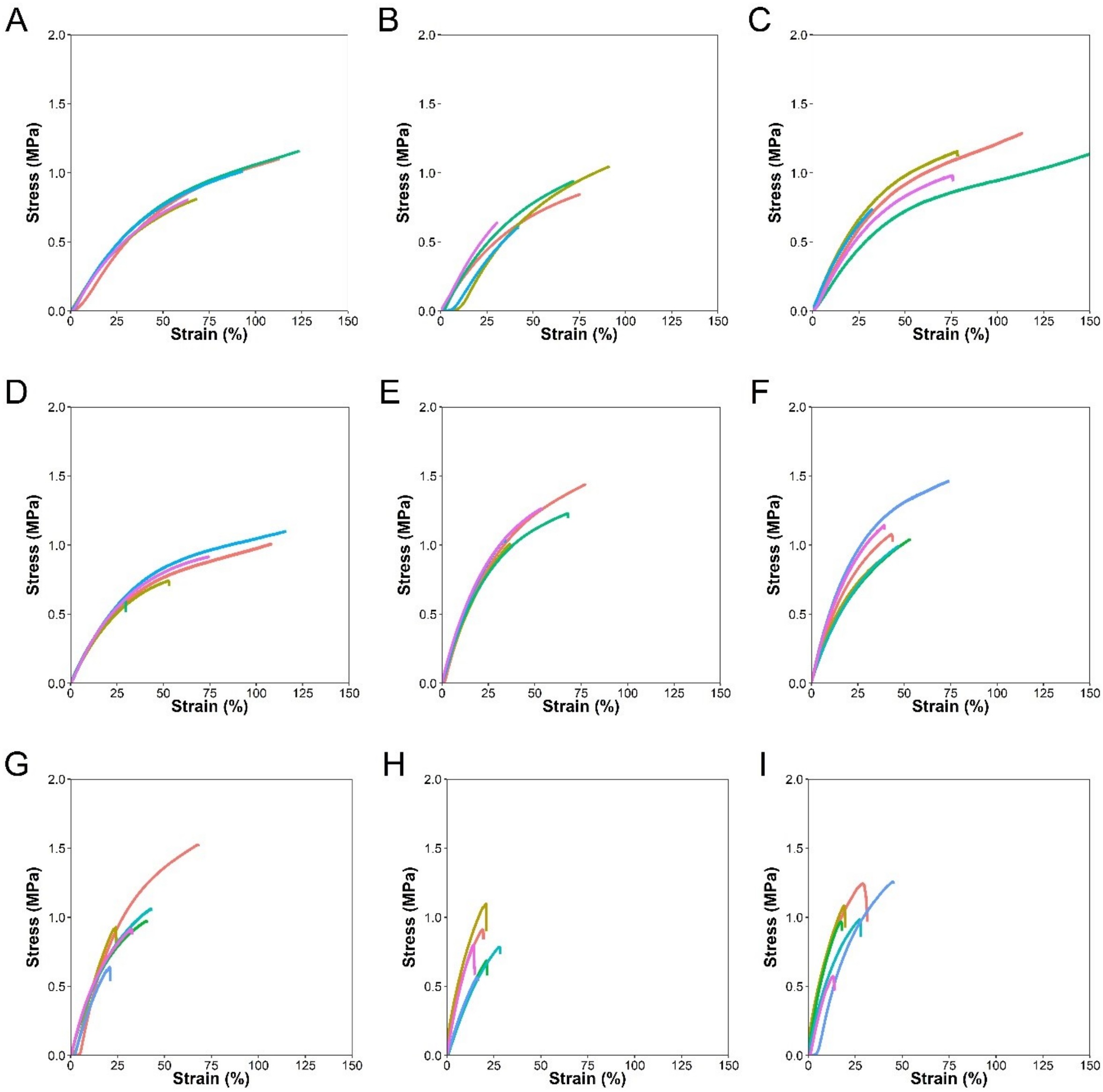

Figure S3. Stress-strain curves for the prepared samples measured in the swollen state in water at room temperature. A) HCNC_00, B) HCNC_01, C) HCNC_03, D) HCHC_06, E) HCNC_09, F) HCNC_12, G) HCNC_15, H) HCNC_18, I) HCNC_22. All samples were measured at 10 mm min^{-1}. (Mean of n = 6 samples ± SD).

Table S2. Mechanical test results for the prepared samples measured in the swollen state in water at room temperature. (Mean of n = 6 samples ± SD).

Composition	Stress-at-break (MPa)	Strain-at-break (%)	Young's Modulus (MPa)	Toughness (MJ m^{-3})
00	0.98 ± 0.16	92.08 ± 26.50	1.21 ± 0.03	0.60 ± 0.28
01	0.81 ± 0.19	62.11 ± 25.06	1.43 ± 0.37	0.31 ± 0.19
03	1.14 ± 0.31	103.55 ± 70.33	1.48 ± 0.46	0.85 ± 0.74
06	0.87 ± 0.20	76.18 ± 36.32	1.32 ± 0.23	0.50 ± 0.33
09	1.20 ± 0.18	53.82 ± 18.83	2.33 ± 0.52	0.44 ± 0.23
12	1.10 ± 0.20	49.12 ± 13.22	2.24 ± 0.32	0.37 ± 0.20
15	1.01 ± 0.29	38.30 ± 16.93	2.59 ± 0.38	0.26 ± 0.21
18	0.81 ± 0.18	20.27 ± 4.74	3.36 ± 0.56	0.10 ± 0.04
22	1.02 ± 0.25	26.04 ± 11.49	3.51 ± 0.89	0.18 ± 0.11

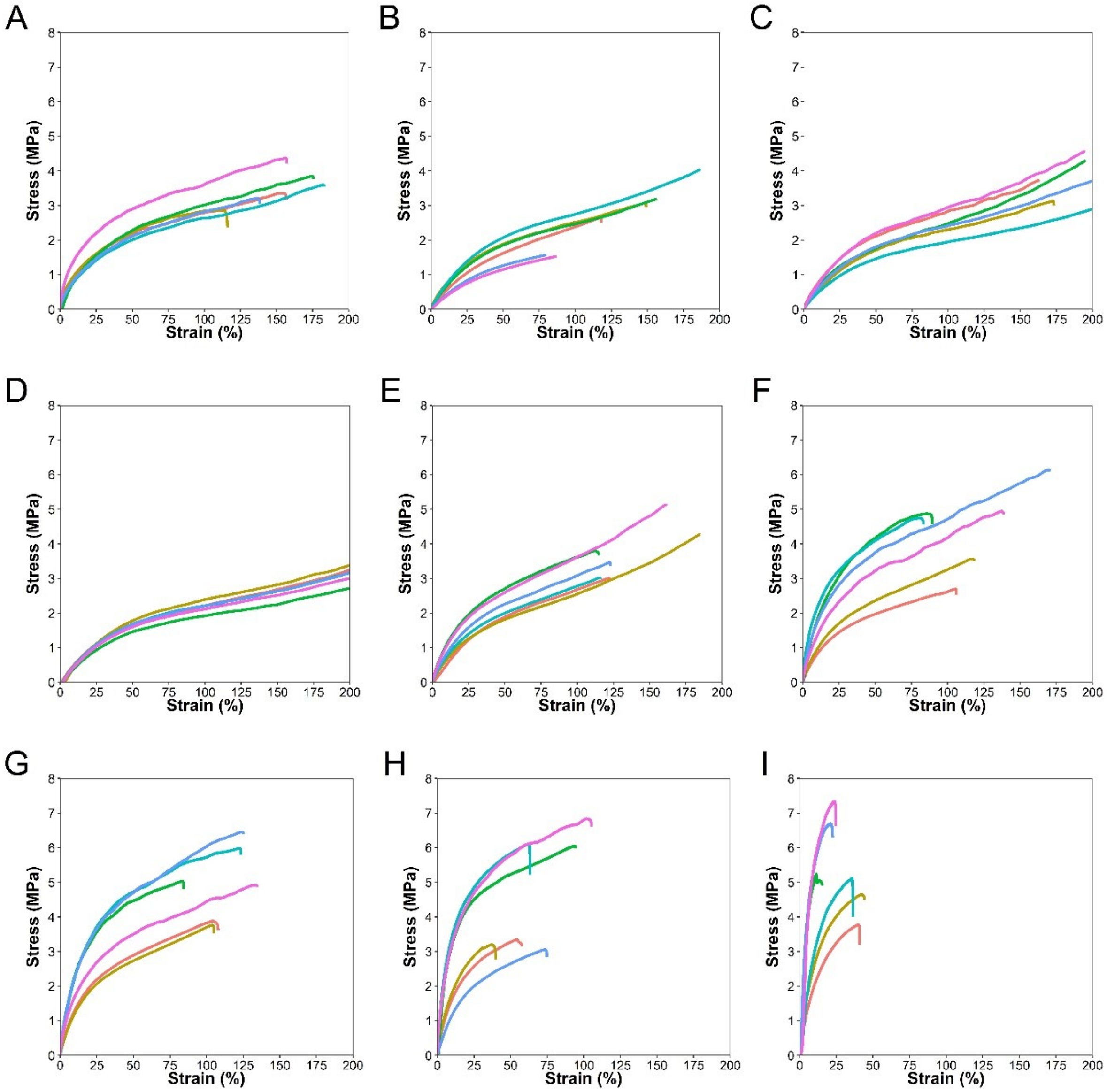

Figure S4. Stress-strain curves for the prepared samples measured in the swollen state in n-hexane at room temperature. A) HCNC_00, B) HCNC_01, C) HCNC_03, D) HCHC_06, E) HCNC_09, F) HCNC_12, G) HCNC_15, H) HCNC_18, I) HCNC_22. All samples were measured at 10 mm min^{-1}.

Table S3. Mechanical test results for the prepared samples measured in the swollen state in n-hexane at room temperature. (Mean of n= 6 samples ± SD).

Composition	Stress-at-break (MPa)	Strain-at-break (%)	Young's Modulus (MPa)	Toughness (MJ m^{-3})
00	3.54 ± 0.53	154.41 ± 24.59	3.42 ± 0.42	3.89 ± 0.96
01	2.67 ± 0.98	129.00 ± 42.02	2.55 ± 0.54	2.44 ± 1.54
03	4.00 ± 0.53	205.02 ± 40.47	2.84 ± 0.40	4.96 ± 1.07
06	3.67 ± 0.25	245.17 ± 10.18	2.51 ± 0.17	5.60 ± 0.42
09	3.78 ± 0.82	137.09 ± 28.99	3.42 ± 0.55	3.35 ± 1.16
12	4.50 ± 1.20	117.77 ± 32.71	4.80 ± 1.26	3.83 ± 1.91
15	5.01 ± 1.08	113.33 ± 18.10	5.58 ± 1.23	4.23 ± 1.42
18	4.77 ± 1.74	72.50 ± 24.18	6.56 ± 1.71	2.85 ± 1.89
22	5.47 ± 1.32	30.66 ± 11.44	19.23 ± 10.80	1.15 ± 0.32
